# Chimpanzees demonstrate individual differences in social information use

**DOI:** 10.1007/s10071-018-1198-7

**Published:** 2018-06-19

**Authors:** Stuart K. Watson, Gillian L. Vale, Lydia M. Hopper, Lewis G. Dean, Rachel L. Kendal, Elizabeth E. Price, Lara A. Wood, Sarah J. Davis, Steven J. Schapiro, Susan P. Lambeth, Andrew Whiten

**Affiliations:** 1 Centre for Social Learning and Cognitive Evolution, and Scottish Primate Research Group, School of Psychology and Neuroscience, University of St Andrews, St Andrews, UK; 2 Department of Veterinary Sciences, National Center for Chimpanzee Care, Michale E. Keeling Center for Comparative Medicine and Research, UT MD Anderson Cancer Center, Bastrop, TX USA; 3 Lester E. Fisher Center for the Study and Conservation of Apes, Lincoln Park Zoo, Chicago, IL 60614 USA; 4 Department of Anthropology, Centre for the Coevolution of Biology and Culture, Durham University, Durham, UK; 5 Centre for Behaviour and Evolution, Institute of Neuroscience, Newcastle University, Newcastle upon Tyne, UK; 6 Division of Psychology, Abertay University, Bell Street, Dundee, UK; 7 Department of Experimental Medicine, University of Copenhagen, Copenhagen, Denmark

**Keywords:** Chimpanzee, Culture, Social learning, Individual differences, Meta-analysis, Sex difference

## Abstract

Studies of transmission biases in social learning have greatly informed our understanding of how behaviour patterns may diffuse through animal populations, yet within-species inter-individual variation in social information use has received little attention and remains poorly understood. We have addressed this question by examining individual performances across multiple experiments with the same population of primates. We compiled a dataset spanning 16 social learning studies (26 experimental conditions) carried out at the same study site over a 12-year period, incorporating a total of 167 chimpanzees. We applied a binary scoring system to code each participant’s performance in each study according to whether they demonstrated evidence of using social information from conspecifics to solve the experimental task or not (Social Information Score—‘SIS’). Bayesian binomial mixed effects models were then used to estimate the extent to which individual differences influenced SIS, together with any effects of sex, rearing history, age, prior involvement in research and task type on SIS. An estimate of repeatability found that approximately half of the variance in SIS was accounted for by individual identity, indicating that individual differences play a critical role in the social learning behaviour of chimpanzees. According to the model that best fit the data, females were, depending on their rearing history, 15–24% more likely to use social information to solve experimental tasks than males. However, there was no strong evidence of an effect of age or research experience, and pedigree records indicated that SIS was not a strongly heritable trait. Our study offers a novel, transferable method for the study of individual differences in social learning.

## Introduction

Overzealous copying of one’s peers may lead to the adoption of sub-optimal (e.g. an inefficient foraging method) or irrelevant behaviours (e.g. a male copying a female courtship gesture) (Giraldeau et al. 2002; Kendal et al. 2005). It has, therefore been proposed that in order for social learning to be adaptive, copying behaviour is guided by ‘transmission biases’ in social learning (Boyd and Richerson 1985; Laland 2004) that influence when individuals use social information and from whom it is best sourced. To date, research on these transmission biases has primarily focussed on model-based biases (e.g. ‘copy dominant individuals’) or contextual-based biases (e.g. ‘copy when uncertain’) (Rendell et al. 2011; Kendal et al. 2015; Price et al. 2017; Watson and Whiten 2017). However, recently there has been an increasing interest in individual differences in social information use that may complement contextual variation (Mesoudi et al. 2016). Optimal learning behaviour is likely to vary between individuals depending on their circumstances (e.g. rapidly changing versus stable habitat), and even within individuals across their development (Wood et al. 2013). Consequently, we would predict that social learning behaviour is somewhat flexible to accommodate such different needs. For example, Farine et al. (2015) experimentally elevated stress hormones in some zebra finch nestlings, but not their siblings. Control siblings followed the developmentally typical bias of copying parental behaviour, whereas hormone-elevated individuals exclusively copied unrelated adults. This demonstrates how even genetically similar individuals may adopt very different social learning behaviour based on their developmental history. We may similarly expect that some individuals are more or less likely to use social, rather than asocial, information in general depending on their life history.

Mesoudi et al. (2016) eschew broad claims about species-typical social learning habits (e.g. ‘species X displays imitation’) and instead advocate greater attention to inter-individual variation in social learning. One approach that has proven effective is to examine whether performance on social learning tasks corresponds with particular ‘personality’ traits, behavioural differences that are stable over time and contexts (Carere and Maestripieri 2013). For example, Carter et al. (2014) measured personality traits in a population of wild baboons and then presented them with two foraging tasks in which individuals could learn from experienced demonstrators. It was found that ‘bold’ and ‘anxious’ individuals reliably showed the most improved performance after observing demonstrations.

In the current study, we present a novel meta-analytical method of examining individual differences in proclivity for using social information from conspecifics to solve experimental tasks. Just as many accumulated years of observational research at field sites have eventually allowed detailed longitudinal analyses of cultural behaviour in wild populations of animals, such as apes, monkeys, marine mammals and meerkats (Santorelli et al. 2011; Allen et al. 2013; Perry et al. 2003; van Schaik et al. 2003; Robbins et al. 2016; Thornton et al. 2010; Whitehead and Rendell 2014; Whiten et al. 1999), a similar accumulation of experimental data has now accrued at some captive research sites. This presents an unprecedented opportunity to examine individual performance of the same individuals across many studies. The National Center for Chimpanzee Care in Texas is one such site, where 16 experimental studies (one unpublished) have investigated social learning over a 12-year period (Davis et al. 2016; Dean et al. 2012; Hopper et al. 2007, 2008, 2012, 2015; Kendal et al. 2015; Price et al. 2009; Vale et al. 2014, 2017c; Watson et al. 2017a, 2018; Whiten et al. 2007; Wood 2013, thesis available at http://www.etheses.dur.ac.uk/7274). We collated these data to investigate whether chimpanzees demonstrate individual differences in their propensity for using social information from conspecifics to solve experimental problems and if so, which characteristics may covary with this propensity. We have directed this investigation in accordance with findings of prior research, introduced below, which are suggestive of effects of rearing history, age and sex on social learning to determine whether such factors predict whether individuals in our study population used social learning in experimental tasks. These factors had the additional benefit that they could be reliably extracted from existing datasets.

Early-life environmental differences can have a profound influence on the social learning behaviour of primates (Bard and Leavens 2014). For instance, chimpanzees that have been raised by humans (so-called ‘enculturated’ chimpanzees) have been found to be more likely to imitate behaviours demonstrated by humans than mother-reared individuals (Bering et al. 2000; Bjorklund and Bering 2003; Buttelmann et al. 2007; Leavens et al. 2017; Furlong et al. 2008). Although it should be noted in the case of Furlong et al. (2008) that enculturation took place after the individuals had spent their first year or two in a nursery setting. However, it has not been established whether the effects of enculturation extend to a greater reliance on social information in general, or just that the preferred mechanism (e.g. imitation versus emulation) of social learning is different in enculturated individuals. It should also be noted that non-enculturated chimpanzees also have imitative capabilities (Whiten and Custance 1996, but see; Tennie et al. 2012).

In addition to the developmental effects described above, there is limited evidence for sex differences in chimpanzee social learning. Lonsdorf (2005) found that wild female juvenile chimpanzees (from 1 to 11 years old) spent more time watching their mother termite-fishing than do their male counterparts and, consequently, the females fished more often, more successfully and mastered the technique on average 27 months earlier than males. The effect of this early difference in behaviour appears to result in persistent differences in foraging methods, as females engage in termite-fishing more often than males in adulthood (McGrew et al. 1979), demonstrate greater proclivity and skill for the difficult process of opening coula nuts using stone tools (Boesch and Boesch 1981) and are more likely to engage in tool-assisted hunting (Pruetz and Bertolani 2007; Pruetz et al. 2015).

Several studies have applied batteries of cognitive tests to large numbers of humans and apes (including chimpanzees) to investigate between- and within-species differences in physical and social cognition. While these studies detected intra-specific influences of age (Lacreuse et al. 2014), sex (Herrmann et al. 2007, 2010) and enculturation (Russell et al. 2011) on performance in some domains of chimpanzee behaviour, including aspects of social cognition, there was limited focus on social learning specifically. Furthermore, social information was always provided by a human experimenter rather than another chimpanzee, limiting the extent to which these findings can be generalised to conspecific interactions.

There is evidence that chimpanzees in early life may be particularly sensitive to social information (Biro et al. 2003; Lonsdorf 2005). For example, Biro et al. (2003) found evidence that chimpanzees have a ‘critical period’ (between 3 and 5 years of age) during which to socially learn the challenging skill of nut-cracking behaviour. If this does not occur, then such individuals are extremely unlikely to master the skill later in life (Biro et al. 2003). This enhanced early-life sensitivity to social information is further evidenced by Lacreuse et al.’s (2014) finding that older female chimpanzees perform worse on social cognition tasks than younger individuals. Whether wild chimpanzees acquire the bulk of their cultural repertoire during this early juvenile period, or if it only affects the acquisition of highly technical skills, such as nut-cracking (just as humans ‘grow out of’ being able to learn a language with ease), remains unclear. However, numerous studies have identified social learning in adult individuals (e.g. Whiten et al. 2005; Watson et al. 2017; Kendal et al. 2015) demonstrating that social learning occurs throughout chimpanzee lifespans (for a review, see Whiten and van de Waal 2018). Moreover, it should also be noted that a chimpanzees’ age has been found not to correlate with success in problem-solving in non-social contexts (Hopper et al. 2014).

As described, a chimpanzee’s sex, rearing history and age are three factors that appear to influence social information use in specific paradigms. Moreover, Thornton and Lukas (2012) found that these same factors were important in explaining individual variation in performance in physical cognition-based tasks across a number of species. We have, therefore, focussed on these variables to determine whether they may also explain individual variation in chimpanzees’ social information use to solve experimental problems. While the rank of an individual chimpanzee has been found to influence their use of social information (Kendal et al. 2015), we did not have longitudinal hierarchy data, so were unable to include this in our analysis. It would also have been interesting to incorporate personality data into our analysis, but this was not available at the time of writing. With regards to rearing history, we categorised individuals as to whether they were mother-reared and born in captivity, nursery-reared and born in captivity, or wild-born. While nursery-reared individuals were not enculturated in that they were not raised in a human home, it is possible that increased interaction with humans during infancy, relative to mother-reared individuals, might cause similar behavioural differences. For example, it has been found (Clay et al. 2017) that chimpanzees reared in a nursery setting exhibited more human-oriented behaviours than mother-reared individuals, although nursery-reared males were also found to behave more aggressively towards humans than mother-reared males. Alternatively, impoverishment of interaction with their mothers may negatively influence proclivity for social learning in chimpanzees. Chimpanzee mothers actively nurture species-typical communicative, social and motor skills (Bard 1994) and the absence of nurturing care can result in abnormalities in grey-matter volume in the basal forebrain (Bard and Hopkins 2018). However, a relationship between these effects on brain structure and social learning behaviour has not been directly examined.

Matrilineal relationships seem to be critical for cultural transmission in several species; for example, communicative signals in chimpanzees (Taglialatela et al. 2012), vocalisations in hump-back whales (Yurk et al. 2002) and food-cleaning techniques in vervet monkeys (van de Waal et al. 2012). Since the parentage of most individuals within the study population was known and multiple individuals from the same family units were present in the population, we also investigated heritability (similarity between related individuals, see Wilson et al. 2010) in propensity to use social information in experimental tasks. Genetic inheritance of a proclivity for social learning has been identified in fruit flies (Foucaud et al. 2013) but is otherwise underexplored. Finally, an important consideration for many scientists choosing their sample is that individuals with a long history of participating in research may behave differently to less experienced peers (e.g. reduced neophobia, or transference of aptitude between tasks). Consequently, we also explored whether the number of social learning studies in which individuals had participated influenced the likelihood that they would use social information in the next study.

## Methods

### Study site

Participants were 167 (76 male) chimpanzees housed at the National Center for Chimpanzee Care located at the Michale E. Keeling Center for Comparative Medicine and Research of The University of Texas MD Anderson Cancer Center in Bastrop, Texas, USA. This site was chosen because, to our knowledge, it has the greatest number of chimpanzees who have participated in such a large number of successive social learning studies.

In 2005, when the earliest data included here were collected, the median age was 20 years old (range 3–43). In 2016, when the final data were collected, the median age was 31 (range 14–51). An important distinction in this analysis was between mother-reared and nursery-reared individuals (Table 1). Nursery-reared individuals were chimpanzees who had been separated from their mother at birth due to abandonment, incompetency or health complications that put their lives at risk. While nursery-reared individuals were housed with conspecific peers when they were old enough to move, they also received relatively large amounts of human contact (approximately 1 h/day) compared to mother-reared individuals for the first few years of life.

**Table 1:** Summary table of rearing history and birthplace of subjects

	Mother	Nursery	Unknown	Total
Wild	42	0	0	42
Captive	97	25	2	124
Unknown	0	0	1	1
Total	139	25	3	167

### Data collation

We contacted all researchers who had carried out studies related to social learning at the study site between the years of 2005 and 2016. In each case we requested:

- A summary of the methods used in the study
- A list of all participants used in all conditions
- Detailed response measures for each participant
- The date range over which data were collected

This resulted in a dataset comprised of 16 studies (Table 3). This included data from a total of 167 individuals who had participated in at least 1 (mode = 3, median = 3) experimental condition (Table 2). Only conditions in which individuals were exposed to either a live or video demonstration by a conspecific were included (i.e. no asocial controls, no human-led training, nor ‘ghost’ conditions, see Hopper et al. 2015).

**Table 2:** Breakdown of the number of chimpanzees who participated in a given number of experiments

*N* experiments	*N* participated
1	18
2	28
3	40
4	34
5	18
6	18
7	9
8	2

Greatest number of experimental participations by any individual was 8. Total = 167

**Table 3:** List of studies used, date data collection commenced and SIS criteria. SIS = 1: individual shows convincing evidence of social information use to solve the experimental task. SIS = 0: individual shows no evidence or ambiguous evidence. The same criteria applied to all experimental conditions within a study

Study	Data collection	SIS	Score criteria
Hopper et al. (2015)	09/2005	1	Used the seeded method on their first trial
		0	Either never opened the puzzle-box or did not use seeded method on first trial
Hopper et al. (2007)	02/2006	1	Used the seeded method on their first trial
		0	Either never opened the puzzle-box or did not use seeded method on first trial
Hopper et al. (2008)	04/2006	1	Used the seeded method on their first trial
		0	Either never opened the puzzle-box or did not use seeded method on first trial
Hopper et al. (2012)	05/2006	1	Used the seeded method on their first trial
		0	Either never opened the puzzle-box or did not use seeded method on first trial
Whiten et al. (2007)	06/2006	1	Learned seeded method of opening a puzzle-box
		0	Did not learn seeded method
Dean et al. (2012)	06/2007	1	Reached at least ‘level one’ of opening a three-stage puzzle-box
		0	Did not reach level one
Kendal et al. (2015)	10/2007	1	Used the seeded method on their first trial
		0	Either never opened the puzzle-box or did not use seeded method on first trial
Price et al. (2009)	04/2008	1	Scored = > 11 on the score used to measure similarity of tool-combination behaviour to that of model
		0	Scored < 11 on the tool-combination score
Vale et al. (2014)	03/2010	1	Ate at model-demonstrated resource-rich location
		0	Ate at model-demonstrated resource-poor location
Wood et al. (unpublished)	05/2011	1	Solved problem after observing demonstration
		0	Never solved
Vale et al. (2017a)	04/2012	1	More than 75% of model-demonstrated alternative tokens exchanged
		0	Less than 75% of model-demonstrated alternative tokens exchanged
Vale et al. (2017b)	03/2015	1	Ate previously unpalatable, group-preferred food > 25% of the time
		0	Ate previously unpalatable, group-preferred food < 25% of the time
Vale et al. (2017c)	06/2015	1	Learned the tool-use sequence in phase 1 or 2
		0	Never learned the tool-use sequence or learned in phase 3
Davis et al. (2016)	04/2015	1	Switched to observed alternative method in Experiment 1
		0	Did not switch to observed alternative method in experiment 1
Watson et al. (2017)	06/2015	1	Used the seeded method on their first trial
		0	Either never opened the puzzle-box or did not use seeded method on first trial
Watson et al. (2018)	06/2016	1	Switched to observed alternative method
		0	Never switched to observed alternative method

To make meaningful comparisons between studies with a disparate array of methodologies, it was necessary to standardise the outcomes as far as possible. In a ‘classic’ meta-analysis this would be done by drawing effect sizes from each of many studies, each using different samples, to identify the overall effect of a given variable. For example, Cross et al. (2011) investigated the influence of sex across a number of measures of impulsivity. However, this approach was not possible in the present study where most of our individuals (149 out of 167, see Table 2) were sampled multiple times across different studies. Consequently, we created a binary scale, applied to the results of each study, which assigned a Social Information Score (SIS) to each individual. A SIS of 0 indicated that an individual showed no evidence or ambiguous evidence of social learning from conspecifics in solving the experimental task. A SIS of 1 indicated that the individual demonstrated convincing evidence for social learning by not only solving the novel task, but also using the same method as that demonstrated by the model (Table 3). For example, Watson et al. (2017) employed a simple two-action puzzle-box paradigm in an open-diffusion context. Groups were seeded with a method of opening the box by either a high- or low-ranking model. A score of 0 would indicate that an individual either (a) never successfully opened the box, or (b) first learned to open the box using a method they had not observed, suggesting that they potentially learned the solution asocially. A score of 1 was given to individuals who had observed the seeded behaviour and used it as their first choice of method. The social learning definition used for each study can be found in Table 3. Where possible and appropriate, the criteria used are closely based on those used by the original study. This binary measure of social learning unfortunately meant that we lost granularity in the data associated with each study. However, this was preferable to the degree of subjective assessment that would be required for a more nuanced scale and which would have rendered cross-study comparisons less meaningful. The application of the SIS criteria listed in Table 3 generated 607 data-points across 26 study conditions and 167 individuals. See Table S1 for a full matrix of study scores and participation for each individual.

Conditions within studies often differed considerably in the methods used, and so each condition within a study was treated as separate for the purposes of the random effect ‘Condition’. Ages of individuals at the time of study were calculated by deducting their date of birth from the approximate date at which data collection for a study began. Pedigree data were also collated for each chimpanzee to determine relatedness between individuals. This allowed us to measure the effect of genetic relatedness on our outcome measures.

### Analysis

To determine which factors were common to individuals who were most likely to score ‘SIS = 1’, we fitted a series of binomial (probit link function) generalised linear mixed-models with random-intercepts (but not slopes, due to the associated difficulty of extracting repeatability measures) using a Bayesian framework. This was carried out using ‘RStudio’ (R Studio Team 2015) and ‘R’ (R Development Core Team 2016) with the package ‘MCMCglmm’ (Hadfield 2010). This package allows the use of pedigree data to estimate the genetic heritability (*h*^2^) of a given trait, a type of analysis known as an ‘animal model’ (Wilson et al. 2010). Values of *h*^2^ that are close to 0 indicate that there is a negligible effect of pedigree (very little similarity in SIS due to relatedness), whereas values close to one indicate a strong effect. For example, if closely related individuals perform more similarly than distantly related or unrelated individuals then we would predict a high value of *h*^2^. MCMC chains were run for 5,000,000 iterations, with a burn-in period of 100,000 iterations and a thinning interval of 1000 iterations to reduce autocorrelation. All models were fit with uninformative priors (*V* = 1, *n* = 0.002) and the residual variance was fixed to 1 because this cannot be estimated when using a binary response variable. Convergence was assessed visually using trace plots of posterior distributions and acceptably low levels of autocorrelation were ensured by determining that all estimated parameters had an effective sample size of over 1000.

We ran two models; a ‘Full’ model containing all possible fixed effects and a ‘Null’ model containing no fixed effects Table 4 details the fixed and random effects present in each model. Repeatability (the proportion of variance in SIS explained by the association of data points with a specific individual, hereafter referred to as ‘individual identity’) was calculated by dividing the variance explained by the random effect for individual by the total variance in SIS (Nakagawa and Schielzeth 2010). Evidence for a strong effect of a variable was determined according to whether the 95% credibility intervals of the posterior distribution (the distribution within which there is a 95% probability that the population mean lies, van de Schoot et al. 2014) crossed zero. If a variable has a negligible effect, we expect its posterior distribution to be centred close to zero. An influential variable is expected to be shifted away from and not substantially overlapping zero. The best-fitting model was determined using an information theoretic approach (Burnham and Anderson 2003). A deviation information criterion (DIC) was derived for each model, from which total DIC weights the probability that a given model is the best fit relative to those in the set, (Wagenmakers and Farrell 2004) for each model were calculated. The full dataset used in this analysis is available in the Open Science Framework repository and can be accessed at: http://www.osf.io/twjzg. For an accessible introduction to terminology and inference in Bayesian statistics, we recommend van de Schoot et al. (2014).

**Table 4:** Fixed effects, random effects and outcome variable used in our models

Fixed effects	
Age	Age (years) of the individual at the time of a given experimental condition
Sex	Sex of the individual
Rearing	Whether individual was captive-born and mother-reared, captive-born and nursery-reared, or wild born
Experience	The number of experimental conditions which the individual had participated in prior to the study in question
Random effects	
Individual identity	Identity code corresponding to each unique individual in the sample, to control for multiple observations and calculate repeatability
Pedigree	An individual’s parentage, if known. Used to estimate heritability of SIS
Condition	The experimental condition (*N* = 26) the data point was taken from
Outcome	
SIS	A binary measure of social learning for a given experimental condition

## Results

**Fig. 1:**
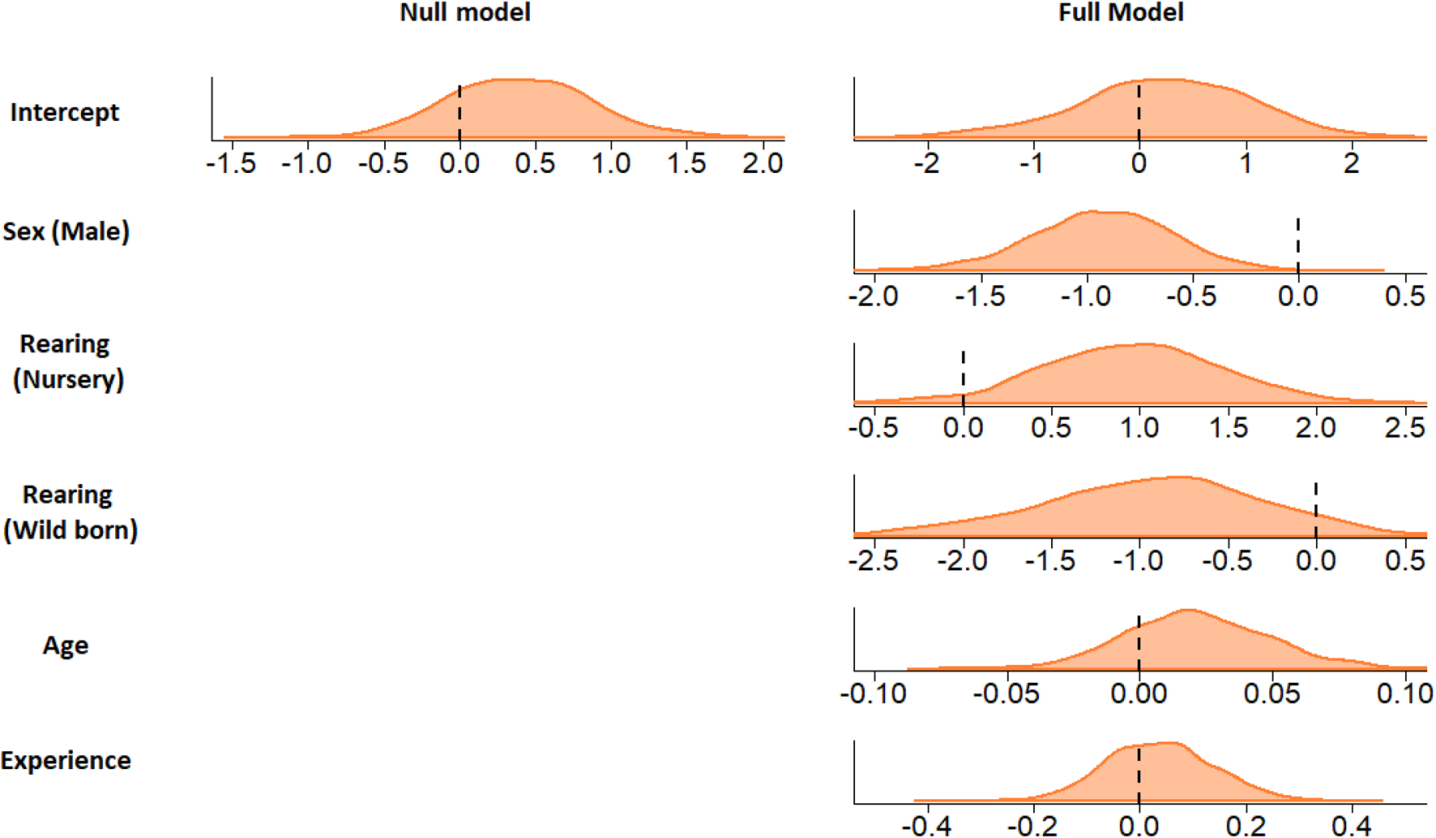
Posterior density distribution plots for each parameter in each model. Sex (male) is relative to sex (female) and rearing (nursery/wild born) is relative to rearing (captive-born, mother-reared)

The full model was found to fit the data substantially better than the null model (SIS ~ ID + pedigree + condition) model, with a total delta information criterion (DIC) weight (probability that this model is the best fit for the data) of 96% (Table 5). In both the full and null model, individuals demonstrated moderate repeatability in SIS (full model: repeatability = 0.52, 95% CI 0.129, 0.734. Null model: repeatability = 0.571, 95% CI 0.246, 0.744. Maximum possible repeatability is 1.0), with individual identity accounting for approximately half of the total variation in SIS. Because there was evidence of a sex difference in SIS for the full model, we calculated the inverse logit of the coefficients to obtain the estimated posterior probability that individuals would use social information to solve the task based on their sex (Table 7), finding that females were estimated to be, on average, 15–24% more likely to use social information than males, depending on their rearing history (Table 7). However, 95% credibility intervals were wide in all cases, indicating a high degree of uncertainty in the models due to variation between individuals within the same sex/rearing category. No other fixed effects in either model had 95% CIs which did not cross zero, indicating that SIS had no strong relationship with the other variables we tested. However, there is also a moderate but highly variable effect of rearing history since their 95% credibly intervals only narrowly cross zero (Fig. 1; Table 6). Heritability was found to have an extremely weak effect, with the posterior estimate of *h*^2^ being less than 0.01 in all models.

**Table 5:** Information criterion and conditional *R*^2^ (all fixed and random effects) statistics with 95% credibility intervals for each model

Model	DIC	Total DIC weight	Conditional *R*^2^
Null	613.77	0.04	0.50 (0.22, 0.73)
Full model	607.25	0.96	0.54 (0.31, 0.75)

**Table 6:** Full summary outputs for each model

Model	Fixed effect	Posterior mean	Lower 95% CI	Upper 95% CI	R^2^ for fixed effect
Null	Intercept	0.390	− 0.565	1.392	–
Full model	Intercept	0.22	− 1.387	2.000	–
	Sex (male)	− 0.909	− 1.563	− 0.343	0.206
	Rearing (nursery)	0.946	− 0.092	1.937	0.086^a^
	Rearing (wild-born)	− 0.888	− 2.237	0.344	0.086^a^
	Age	0.021	− 0.033	0.073	0.048
	Current experience	0.037	− 0.166	0.227	0.004

^a^*R*^2^ applies to fixed effect as a whole, not individual levels

**Table 7:** Estimated posterior probability of that an individual will score SIS = 1 in any given study by sex and early-life history (full model)

Sex	Rearing	Probability SIS = 1	Lower 95% CI	Upper 95% CI
M	Mother	0.329	0.086	0.725
F	Mother	0.561	0.200	0.881
M	Nursery	0.551	0.185	0.902
F	Nursery	0.798	0.318	0.953
M	Wild-born	0.155	0.015	0.707
F	Wild-born	0.308	0.038	0.870

## Discussion

This study used data collated from the same study site across 26 experimental conditions from 16 different studies of social learning, to explore whether sex, rearing history, age, prior research experience and genetic heritability had an important effect on chimpanzees’ proclivity for using social information from conspecifics to solve experimental problems. Of these factors, we identified a sex difference in SIS, with female individuals being 15–24% more likely to use social information to solve experimental problems than males, depending on their rearing history (Table 7). The data also suggests that rearing history may have an influence, but that there is a great deal of variation within each category. However, there was no evidence that age at the time of a study, nor the number of social learning studies an individual had participated in prior to a given study had a strong effect on SIS. Furthermore, there was little indication in these data to suggest that proclivity for social learning is a heritable trait, unlike performance in paradigms designed to test ‘general’ intelligence (Hopkins et al. 2014).

Repeatability estimates found that approximately half of the variance in SIS was explained by individual identity in each model. This indicates that individual differences play an important role in the transmission of social information in chimpanzees. Given that sex, the only other factor found to have a strong effect on SIS, accounted for less than half of the variance explained by individual identity, other stable features of an individual, such as personality (King et al. 2008), are likely to be worth exploring to account for the missing variance. There is some precedent for this approach, as a relationship between ‘exploratory’ personalities and social information use has been found to be positive in some avian and piscine species (Marchetti and Drent 2000; Nomakuchi et al. 2009). Furthermore, wild baboons that were scored most highly on ‘boldness’ or ‘anxious’ traits were found to show a greater improvement on a foraging task after observing a demonstrator (Carter et al. 2014). In addition, Hopper et al. (2014) found that certain chimpanzee personality traits (‘methodical’, ‘openness’ and ‘dominance’) are associated with success in asocial learning paradigms. Examining personality in parallel with performance across a broad range of social learning tasks would be useful in exploring how proclivity for social information use fits into the broader tapestry of chimpanzee personality traits.

Our analysis suggests that rearing history also has some effect on social information use, though there was a large degree of variability within each category (Fig. 1; Table 6). Relative to captive-born, mother-reared individuals, nursery rearing had a positive relationship, whereas being wild-born had a negative relationship with social information use. Prior literature found human-raised chimpanzees more readily engaged in human-directed imitative behaviour (Bering et al. 2000; Bjorklund and Bering 2003; Buttelmann et al. 2007; Tomasello et al. 1993). The fact that there was no evidence of a robust effect of nursery-rearing in our analysis may reflect that, while our nursery-reared individuals were exposed to significant amounts of human contact compared to mother-reared individuals, they were not raised within a human family, as with the studies cited above. Consequently, although these individuals were accustomed to human interactions, they were not truly enculturated. Engagement with experimental tasks proximate to humans may even be inhibited in some nursery-reared chimpanzees, as Clay et al. (2017) found that nursery-reared males exhibited higher rates of aggressive behaviour towards humans. Additionally, it may be that human contact influences the mechanism of social learning that individuals preferentially deploy (e.g. imitation, see Buttelmann et al. 2007), but not general proclivity for social information use (the broad granularity of effect measured here).

Our finding that females were more likely than males to use social information from conspecifics to solve problems in experimental contexts is consistent with the findings of Lonsdorf (2005) where it was found that young females observed their mothers more and acquired related tool-use competence earlier. A tendency for female chimpanzees to use social information more than males may indicate that males who a greater reliance on asocial learning, as reflected by Reader and Laland’s (2001) finding that a disproportionate number of innovations originate in male chimpanzees. Our finding also potentially sheds further light on the discovery of Lind and Lindenfors (2010) that the number of cultural traits in chimpanzee communities correlates with the number of females, but not males, in the group. If females are more likely to use social information than males, then they are also likely to amass a larger cultural repertoire—the impact of which upon the total group repertoire size is likely to be further increased by the fact that they are the migratory sex (Luncz et al. 2015). However, in chimpanzees, males are typically socially dominant relative to females. Our results could, therefore, reflect the fact that males may typically have priority access to food resources, meaning fewer opportunities for social learning, whereas females potentially observe others before gaining access to the resource.

As previously noted, the studies in our dataset did not examine the influence of rank on task access. The dataset used for this analysis spanned 12 years, during which hierarchies were not systematically recorded but are likely to have shifted within groups and some individuals were moved between groups. Thus, it was not possible to dissociate dominance and sex (or to include an accurate measure of rank in our models). Although these two factors are inexorably tied together, an example of why a distinction might matter comes from Kendal et al. (2015, included in our dataset) who introduced a puzzle-box to 42 chimpanzees living in four social groups (two groups with solutions seeded by trained demonstrators, two without) and observed the diffusion of solutions to this novel foraging problem. It was found that there was a general tendency for individuals to attend to demonstrations from individuals more dominant than themselves. Furthermore, whether or not individuals chose to use social information to open the puzzle-box at all varied greatly according to their own knowledge states and position in the social hierarchy, with more dominant individuals being less likely to attend to the demonstrations of others.

It is somewhat surprising that we did not find evidence that age had an effect upon social learning, given the evidence that young individuals undergo an enhanced “sensitive period” in social learning behaviour (Biro et al. 2003) and elderly individuals decline in social cognition (Lacreuse et al. 2014, although see; Hopper et al. 2014). Perhaps while there may be an early sensitive period in chimpanzees’ proclivity for social learning, once passed, social learning may remain relatively stable over the rest of their lives. Alternatively, it may be that due to the environmental stability characterising captivity relative to the wild, captive individuals may be more stable throughout their lifetime in the behavioural strategies they deploy (such as prioritising social information or not) than wild individuals. We note that since the population was non-reproducing, only 28 of our 607 data points, representing just seven chimpanzees, came from individuals under the age of 10.

There are certain limitations when drawing inferences from the present results. Firstly, although we attempted to standardise the measures used in this analysis across studies, there was no way to objectively control for task difficulty. As a result, individuals who may have participated in five cognitively ‘easy’ tasks (e.g. choosing whether to slide a door left or right, as in Kendal et al. 2015) were judged by the same criteria as those who participated in five ‘difficult’ tasks (e.g. combining tools, as in Price et al. 2009). It is therefore, possible that participants in these latter tasks used social learning, yet were unsuccessful due to a lack of physical dexterity required for the task. For example, it has been found that wild-born captive chimpanzees were more likely to successfully use a tool in a food-raking task than captive-born individuals (Brent et al. 1995). This confound of task difficulty may account for the fact that there was no strong evidence that prior research experience had an effect on SIS in our analysis. Through careful re-coding of the video data associated with each study, behavioural cues of social learning (e.g. model-matching attempts) could possibly be extracted to determine whether individuals were socially learning, but not fully replicating the model’s behaviour (either by not solving the task, or solving it differently). Alternatively, it may be that individuals are more likely to use social information when faced with more difficult experimental tasks, where innovating a solution is likely to be costlier, known as the ‘costly information hypothesis’ (Boyd and Richerson 1985). There is some evidence indicating that other primates behave in a manner consistent with this hypothesis, as Kendal et al. (2009) found that seven species of callitrichid monkeys only used social learning when faced with the most difficult of three foraging tasks. Similarly, in that risk equates to ‘cost’, Brand et al. (2018) found that women are more likely than men to use social information in an experimental task when the asocial option is ‘risky’ (highly variable reward). Women in this context were also more likely to use social information than both men and women in the control condition, where both social and asocial options were non-risky.

Another limitation of the current study was that none of the studies included in the dataset investigated social learning under totally ‘natural’ conditions (e.g. Hobaiter et al. 2014) and involved human experimenters (i.e. were conducted by familiar humans, although all used conspecifics as models, in contrast to others, such as Tomasello et al. 1993). We cannot, therefore, necessarily dissociate proclivity to participate and use social information in the context of experiments (rewarding due to interaction with humans and food rewards) from social information use in general. Less human-oriented individuals who avoid experiments may nevertheless commonly use social information in more everyday contexts. As an example of how human–chimpanzee relationships can influence behaviour, Brosnan et al. (2015) found that human-oriented chimpanzees were found to be more reactive to inequity in food payoffs compared to other individuals. The authors argue that this may have been because the food was distributed by a human, leaving the human-oriented individuals antagonised not only by the reduced payoff but also by the apparent slight from the researcher (see also Engelman et al. 2017).

Despite these limitations, our findings have important implications for how experimenters conducting studies of chimpanzee behaviour choose and balance their sample. For example, when attempting to determine the existence of a hotly contested behaviour pattern such as conformity (Van Leeuwen and Haun 2013; Van Leeuwen et al. 2015), it may be beneficial to sample individuals who are typically most reliant on social learning, to test whether a conformity effect exists at least within such a subset of keen social learners. This way, the likelihood of identifying an extant, but elusive, behaviour is amplified. In contrast, using a sample consisting of individuals who rarely use social information is likely to yield false negatives. Either way, we must then of course be extremely cautious about generalising such samples to wider populations (Mesoudi et al. 2016).

This study applied a novel method of using a ‘meta-data’ set at a chimpanzee research site, incorporating 16 studies carried out over the last 12 years, to examine consistent inter-individual differences in performance across a spread of social learning experiments. It was found that individuals showed marked differences in social information use according to individual identity and sex, with females typically being much more likely to use social information from conspecifics to solve experimental tasks than males. Our methods could, in principle, be applied to any population with a similar scale of data to draw upon.
